# Freeze Drying Improves the Shelf-Life of Conductive Polymer Modified Neural Electrodes

**DOI:** 10.3390/bioengineering2030176

**Published:** 2015-08-07

**Authors:** Himadri S. Mandal, Richard O. Cliff, Joseph J. Pancrazio

**Affiliations:** 1 Blackrock Microsystems, 630 Komas Dr #200, Salt Lake City, UT 84108, USA; 2 System of Systems Analytics, 11250 Waples Mill Road, Fairfax, VA 22030, USA; E-Mail: rcliff@sosacorp.com; 3 Department of Bioengineering, Volgenau School of Engineering, George Mason University, 4400 University Drive, MS 1G5, Fairfax, VA 22030, USA; E-Mail: jpancraz@gmu.edu

**Keywords:** conductive polymer, PEDOT, microwires, impedance, neural electrode

## Abstract

Coating microelectrodes with conductive polymer is widely recognized to decrease impedance and improve performance of implantable neural devices during recording and stimulation. A concern for wide-spread use of this approach is shelf-life, *i.e.*, the electrochemical stability of the coated microelectrodes prior to use. In this work, we investigated the possibility of using the freeze-drying process in order to retain the native low impedance state and, thereby, improve the shelf-life of conductive polymer poly(3,4-ethylenedioxythiophene) (PEDOT)-PSS modified neural electrodes. Control PEDOT-PSS coated microelectrodes demonstrated a significant increase in impedance at 1 kHz after 41–50 days of room temperature storage. Based on equivalent circuit modeling derived from electrochemical impedance spectroscopy, this increase in impedance could be largely attributed to a decrease in the interfacial capacitance consistent with a collapse and closing of the porous structure of the polymeric coating. Time-dependent electrochemical impedance measurements revealed higher stability of the freeze-dried coated microelectrodes compared to the controls, such that impedance values after 41–50 days appeared to be indistinguishable from the initial levels. This suggests that freeze drying PEDOT-PSS coated microelectrodes correlates with enhanced electrochemical stability during shelf storage.

## 1. Introduction

**Figure 1:**
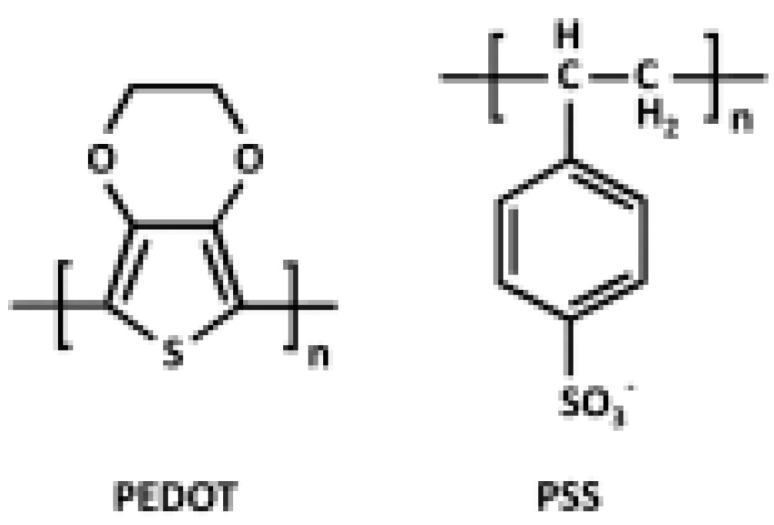
Chemical structures of Poly(3,4-ethylenedioxythiophene) (PEDOT) and Poly(sodium 4-styrenesulfonate) (PSS).

Conductive polymer (CP) modified microelectrodes are promising for developing small but sensitive and powerful neural interfaces for recording and stimulation [1,2,3,4,5,6,7,8]. Because of the porous morphology, CP modified electrodes offer a high degree of electrochemically active surface area and have orders of magnitude lower impedance compared to their metallic counterparts [9,10]. Poly(3,4-ethylenedioxythiophene) (PEDOT), more specifically PEDOT-PSS (Poly(sodium 4-styrenesulfonate)) (Figure 1), is the most extensively studied CP that has been used to modify neural microelectrodes [3,11,12,13,14].

In general, the PEDOT modification of neural microelectrodes is very straightforward and involves electrochemical deposition [3,11,12,13,14]. But a major consideration related to the handling of these modified microelectrodes is the concern that they may not retain their low impedance state under ambient conditions. For this reason, PEDOT electrodes are prepared as needed and quickly used during experimentation. This short shelf-life leads to a severe limitation in terms of storage and/or transportation, and is a bottleneck for a wide spread use of the polymer modified microelectrodes. To date, there is no report in the literature that addresses this issue.

In our previous reports [7,8] on the mechanistic investigation of the solution aging process in PEDOT modified neural electrodes, we demonstrated a direct relationship between the time-dependent increase in the electrode impedance and the gradual decrease in the electrochemically active surface area due to the collapse of the porous polymeric morphology. Based on these observations, we suspected that a similar type of mechanism could also be involved behind the low shelf-life of PEDOT modified electrodes. In addition, based on some reports describing the “capillary-driven” collapse of porous nano-materials after solvent evaporations [15,16,17,18], we hypothesized that the drying process after the solution phase electrochemical PEDOT modification is probably a critical step that requires a thorough investigation.

Freeze-drying, also known as lyophilization, involves the removal of the solvent directly from the solid phase to the vapor phase and has been reported to keep the native structure intact in porous nano-materials [19,20]. Inspired by these findings, we postulated that the capillary-driven collapse could be avoided by freeze-drying PEDOT modified neural electrodes after the electrodeposition. In this work, we show that freeze-drying can indeed significantly increase the stability of PEDOT modified electrodes.

## 2. Experimental Section

### 2.1. Materials

3,4-Ethylenedioxylthiophene (EDOT) and Poly(sodium 4-styrenesulfonate) (PSS) were purchased from Sigma-Aldrich (St. Louis, MO, USA). Phosphate-buffered saline (PBS) was from Mediatech Inc. (Manassas, VA, USA). Pt/Ir implantable microwires (PI20030.1A5) were obtained from MicroProbes for Life Sciences (Gaithersburg, MD, USA).

### 2.2. Preparation of Polymer Modified Implantable Microwires

Cyclic voltammetry (CV) was used to dynamically eletrodeposit [21] the polymer (PEDOT:PSS) on Pt/Ir implantable microwires using a CHI 660D potentiostat (CH Instruments, Inc., Austin, TX, USA) from an aqueous solution (50 mL) of EDOT (0.01 M) with 0.1 M PSS. The potential range was 0–0.75 V *vs.* Ag/AgCl reference electrode (scan rate was 1 V/s; 250 cycles, total charge 3.5 μC).

### 2.3. Freeze-Drying of Polymer Modified Implantable Microwires

After the PEDOT modification, the microwire electrodes were rinsed with PBS, EIS was recorded and quickly freeze-dried using a Virtis Advantage (Gardiner, NY) lyophilizer. The lyophilization protocol was immediate shelf freezing to −40 °C, followed by 12 h of primary drying, with subsequent temperature ramping to 20 °C and 8 additional hours for secondary drying. The freeze-dried microwires were placed in individual vials, flushed with high purity Nitrogen gas, sealed and stored. The control non-freeze-dried PEDOT: PSS modified microwires were similarly flushed with Nitrogen and stored.

### 2.4. Cyclic Voltammetry (CV), Electrochemical Impedance Spectroscopy (EIS) and Modeling

CV and EIS of the modified Pt/Ir microwires were recorded using a potentiostat (CHI 660D, CH Instruments, Inc., Austin, TX, USA) in PBS at 22 °C. The potential range in CV was 0–0.5 V *vs.* Ag/AgCl reference electrode (scan rate was 1 V/s). The impedance was measured at the open circuit potential from 0.1 Hz to 1 MHz, with a peak-to-peak amplitude of the sinusoidal voltage of 20 mV. ZsimpWin (Princeton Applied Research, Oak Ridge, TN, USA) was used to fit the EIS data.

## 3. Results and Discussion

**Figure 2:**
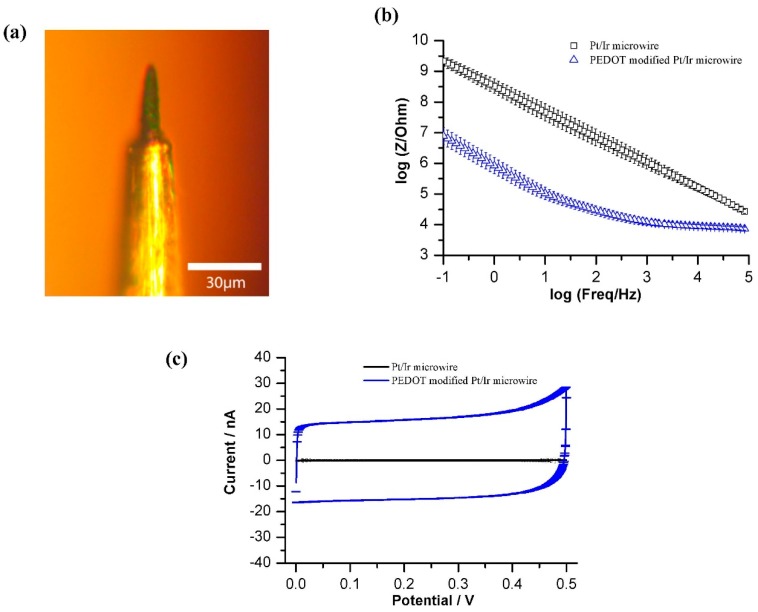
(**a**) Optical image of a PEDOT-PSS modified Pt/Ir microwire electrode; (**b**) Electrochemical Impedance Spectroscopy (EIS) and (**c**) Cyclic voltammetry (CV) (with the coefficient of variation) of microwires in phosphate-buffered saline (PBS) before and after the PEDOT-PSS modification.

A total of 50 commercially available implantable Pt/Ir microwire electrodes were electrochemically modified with PEDOT-PSS. Figure 2b shows the typical impedance of a microwire in PBS before and after the PEDOT modification. The impedance decreased over a wide a range of frequency and the corresponding non-Faradic current in CV (Figure 2c) increased significantly upon the modification which are in line with previous publications, and are related to the high surface area due to porous polymeric morphology [5,6,12,22,23,24]. 25 microwire electrodes were freeze-dried, stored and sealed under N_2_ in individual vials. The control group of 25 non-freeze-dried PEDOT-PSS modified microwires were similarly flushed with N_2_ and stored.

**Figure 3:**
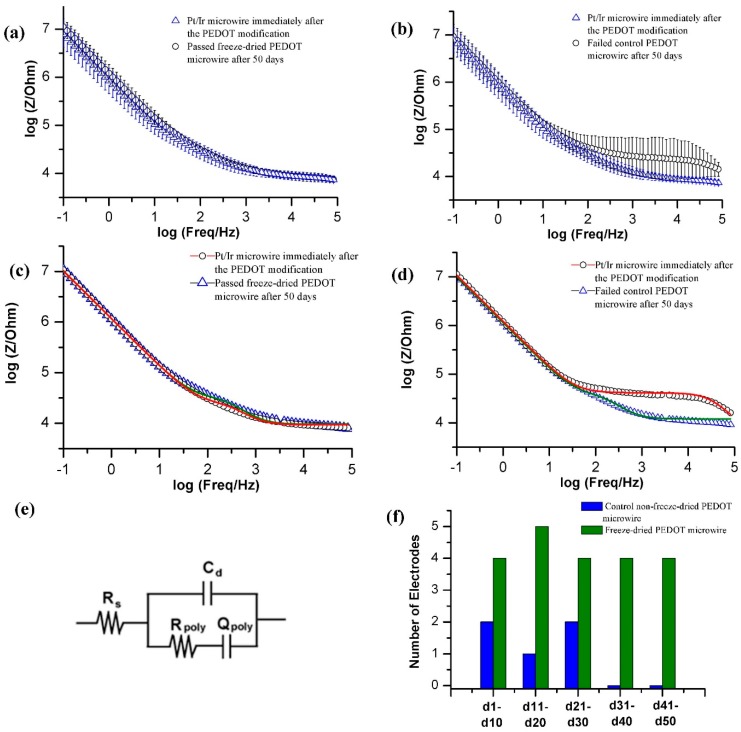
EIS data (with the coefficient of variation) in PBS, immediately after the PEDOT-PSS modification (blue triangle) and after 50 days (black circle) of: (**a**) passed freeze-dried microwires; (**b**) control failed microwires; (**c**,**d**) EIS data from one representative microwire from each group. The red and green solid lines in both figures indicate the respective fit; (**e**) The circuit used to fit the experimental EIS data; (**f**) Stability comparison of freeze-dried *vs*. control non-freeze-dried samples. Figure shows the number of passed microwires with less than 10% change in their original impedance at 1 kHz. The green and blue bars represent the passed freeze-dried and control microwires, respectively, out of 5 in each group.

Every 10 days, 5 freeze-dried PEDOT modified microwires and 5 controls were taken out from sealed vials, placed in PBS, and EIS was recorded. Any electrode showing 10% increase in the impedance at 1 kHz compared to that immediately after electrochemical deposition was considered to be failed. The impedance magnitude at 1 kHz was chosen because of its correspondence to the bandwidth of common electrophysiologic signals associated with neural recording [1,2,12,22]. Freeze-dried PEDOT modified microwires showed higher stability compared to the non-freeze-dried controls (Figure 3f). Note that we also recorded the CV of the PEDOT modified electrodes (Figure S1). However, the change in the non-Faradic current in CV of the corresponding failed electrodes was found to be negligible most probably because of the insensitivity of CV in the 1 kHz regime.

In order to reveal the failure mechanism in the non-freeze-dried PEDOT modified microwires, we fit the EIS data for a failed device (d41–d50) using a previously published [7] electrical equivalent circuit model for CP modified implantable neural microelectrodes (Figure 3e). Here, R_poly_ and Q_poly_ are the charge transfer resistance and the constant phase element or CPE of the associated non-ideal double layer capacitor across the polymeric interface, respectively. R_S_ is the solution resistance and C_d_ is the double layer capacitance due to the pores and/or defect sites that directly expose the metallic electrode surface to the solution. R_poly_ remains similar (Table 1) indicating that the inherent polymer conductivity is not significantly affected which is further supported by the unchanged corresponding Q_poly_. This is in contrast to that observed in solution aging process where we observed a significant increase in the R_poly_ indicating a time dependent polymeric aging [7,8]. However, C_d_ decreases by orders of magnitude for the failed microwire and points to a reduction in the electrochemical surface area. This is consistent with the previously published solution aging process [7,8] and most likely specifies the increase in the impedance due to the collapse and closing of the porous structure. The increase in impedance in the high frequency regime (Figure 2d) may indicate closing of the relatively smaller pores of the polymer structure.

**Table 1:** Fit parameters for the EIS data from a non-freeze-dried microwire immediately after the PEDOT deposition and after failing during d41–d50. Values in the parentheses correspond to the rel. std. error (%). Parameters for a passed freeze-dried sample (d41–d50) are also added for comparison. *n* is the corresponding frequency power necessary for a better fit for non-ideal capacitors.

	Non-Freeze-Dried, Immediately after PEDOT Modification	Non-Freeze-Dried (d41–d50)	Freeze-Dried, Immediately after PEDOT Modification	Freeze-Dried (d1–d50)
R_s_ (Ohm-cm^2^)	1.21 × 10^4^ (5.22 × 10^0^)	4.31 × 10^3^ (2.54 × 10^0^)	9.66 × 10^3^ (2.93 × 10^0^)	9.39 × 10^3^ (2.30 × 10^0^)
C_d_ (F/cm^2^)	2.88 × 10^-8^ (1.50 × 10^1^)	1.41 × 10^-10^ (7.62 × 10^0^)	2.11 × 10^-8^ (7.70 × 10^0^)	2.46 × 10^-8^ (7.17 × 10^0^)
R_poly_ (Ohm-cm^2^)	3.75 × 10^4^ (2.09 × 10^1^)	3.71 × 10^4^ (3.15 × 10^0^)	2.96 × 10^4^ (9.03 × 10^0^)	2.25 × 10^4^ (8.89 × 10^0^)
Q_poly_ (S-sec^n^/cm^2^)	1.24 × 10^-7^ (9.49 × 10^0^)	1.44 × 10^-7^ (2.69 × 10^0^)	1.28 × 10^-7^ (4.53 × 10^0^)	1.27 × 10^-7^ (3.78 × 10^0^)
*n* (0 < *n* < 1)	9.38 × 10^-1^ (2.88 × 10^0^)	9.48 × 10^-1^ (8.34 × 10^-1^)	9.38 × 10^-1^ (1.36 × 10^0^)	9.29 × 10^-1^ (1.07 × 10^0^)

The most likely explanation for the higher stability of the freeze-dried PEDOT modified electrodes is that the freeze-drying process involves a quick initial transition to a frozen solid state and then evaporation of the solvent molecules directly from the solid to a vapor state. This process could essentially bypass the liquid phase related capillary-driven collapse of the porous PEDOT structure. In other words, freeze-drying locks the native structure of the polymer with the high electrochemically active surface area.

## 4. Conclusions

In conclusion, we showed that freeze drying could be a useful method to increase the stability of PEDOT modified neural electrodes. We also demonstrated that the probable mechanism for the failure mode is related to the collapse of the porous polymeric structure, closing of the small pores first and consequent reduction in the electrochemically active surface area. We believe that these findings will facilitate a wider use of CP modified neural electrodes by improving the shelf-life for storage and transportation, and potentially reducing the cost through repeated and long term multiple use. Besides, this may also help other fields where conductive PEDOT films are extensively used, for example, in organic electronics.
